# Total RNA sequencing of *Phlebotomus chinensis* sandflies in China revealed viral, bacterial, and eukaryotic microbes potentially pathogenic to humans

**DOI:** 10.1080/22221751.2022.2109516

**Published:** 2022-08-31

**Authors:** Jing Wang, Qin-yu Gou, Geng-yan Luo, Xin Hou, Guodong Liang, Mang Shi

**Affiliations:** a The Center for Infection & Immunity Study, School of Medicine, Shenzhen campus of Sun Yat-sen University, Shenzhen, People’s Republic of China; b State Key Laboratory of Infectious Disease Prevention and Control, National Institute for Viral Disease Control and Prevention, Chinese Center for Disease Control and Prevention, Beijing, People’s Republic of China

**Keywords:** Sandfly, vector-borne pathogens, virome, total microbiome, meta-transcriptomics

## Abstract

*Phlebotomus chinensis* sandfly is a neglected insect vector in China that is well-known for carrying *Leishmania*. Recent studies have expanded its pathogen repertoire with two novel arthropod-borne phleboviruses capable of infecting humans and animals. Despite these discoveries, our knowledge of the general pathogen diversity and overall microbiome composition of this vector species is still very limited. Here we carried out a meta-transcriptomics analysis that revealed the actively replicating/transcribing RNA viruses, DNA viruses, bacteria, and eukaryotic microbes, namely, the “total microbiome”, of several sandfly populations in China. Strikingly, “microbiome” made up 1.8% of total non-ribosomal RNA and comprised more than 87 species, among which 70 were novel, including divergent members of the genera *Flavivirus* and of the family Trypanosomatidae. Importantly, among these microbes we were able to reveal four distinguished types of human and/or mammalian pathogens, including two phleboviruses (hedi and wuxiang viruses), one novel Spotted fever group rickettsia, as well as a member of *Leishmania donovani* complex, among which hedi virus and *Leishmania* each had > 50% pool prevalence rate and relatively high abundance levels. Our study also showed the ubiquitous presence of an endosymbiont, namely *Wolbachia*, although no anti-viral or anti-pathogen effects were detected based on our data. In summary, our results uncovered the much un-explored diversity of microbes harboured by sandflies in China and demonstrated that high pathogen diversity and abundance are currently present in multiple populations, implying disease potential for exposed local human population or domestic animals.

## Introduction

Arthropod-borne diseases, such as dengue fever, forest fever, malaria, and leishmaniasis, pose a constant threat to public health across the globe, with newly emerging pathogens or variants occurring periodically and causing a greater number of human illnesses and deaths [1]. To keep track of the emerging arthropod-borne pathogens remains a challenging task, mainly because the emerging pathogens are often maintained through enzootic cycles (i.e. between vectors and wild-life animals) before their jump to the human species [2]. Alternatively, they may be originated from pathogens causing mild diseases before a few changes in the genome to make them pandemic strains with concerning clinical manifestation, for which the 2016–2018 epidemic of zika viruses in the Americas is an excellent example [3]. In China, the effort to uncover emerging arthropod-borne pathogens is further hindered by the fact that most of the pathogen surveys have been focused on mosquitos and ticks, whereas limited survey has been carried out on a number of other blood-sucking vectors, such as sandflies, which were collectively referred to as the neglected arthropod vectors [4].

Sandflies (Order Diptera, Subfamily Phlebotominae) are flying, piecing, and blood-sucking vectors well known for transmitting diseases such as leishmaniasis [5] and pappataci fever [6]. The former was caused by a eukaryotic microbe *Leishmania* endemic in over 90 countries and causing > 50,000 deaths per year across the globe [7], whereas the latter was caused by members of the genus *Phleboviruses* mainly reported in countries along the Mediterranean coast [6]. Other pathogens or potential pathogens carried by sandflies included the bacteria from genera *Bartonella* and *Coxiellaceae* [6,8], and viruses from the genera *Vesiculovirus* and *Orbivirus* [9]. In China, the investigations for emerging pathogens in sandflies were mostly limited to *Leishmania* until a recent effort to look into viruses [10–13] and bacteria [8]. Strikingly, two distinctive virus species, wuxiang virus and hedi virus, were isolated using mammalian cell lines and were found to be related to human-infecting members of the genus *Phlebovirus*, suggesting their potential role in causing diseases in mammalian hosts, including humans [10–13]. Despite these discoveries, our knowledge of the general pathogen diversity and overall virome composition for sandflies in China is unclear.

Recently, a whole transcriptome shotgun sequencing approach, namely meta-transcriptomics, has transformed our understanding of the RNA virosphere [14]. To date, more than 10,000 new viral species have been discovered by the meta-transcription approach from a variety of sources, including arthropods [15,16], wild-life mammals [17], non-mammalian vertebrates [18], and a number of environmental sources [19]. In addition to RNA viruses, meta-transcriptomics can also reveal and characterize a wide range of DNA organisms (i.e. DNA viruses, bacteria, and eukaryotic microbes) [20-22] such that it is now the candidate method to simultaneously cover the entire spectrum of microbial organisms, namely, the “total microbiome” [23]. More importantly, the genomic/transcriptomics information uncovered by meta-transcriptomics can be further used to characterize the pathogens, including their origin, evolutionary history, and epidemiological features, and, therefore it presents a powerful tool for modern pathogen discovery works [23].

In this study, we used the meta-transcriptomics approach to survey the “total microbiome” of *Ph. chinensis* sandflies sampled in Shanxi province of China. The object of this study is to first reveal the entire diversity of virome, bacteriome, and eukaryotic microbiome, and from there further analyses were carried out to identify and characterize potential pathogens of humans or other mammalian species. And with that, our data provided a comprehensive evaluation of the disease risk of this neglected arthropod vector.

## Materials and methods

### Sandfly collection and sample processing

From June to July 2019 and 2020, we collected sandflies in four counties of Shanxi Province, China: HeJin, RuiCheng, WuXiang, and XiangNing. Sandflies were sampled using Wentaitai MM200 traps (Guangzhou Changsheng Chemical Technology Service Co, https://www.globalsources.com/si/AS/Guangzhou-Changsheng/6008849913119/Homep-age.htm#) during the night (from 6.00am to 7.00pm next morning) [10]. The collected sandflies were first morphologically identified at the species level and later confirmed by analyzing the cytochrome C oxidase subunit I (COI) gene. The samples were transported to the laboratory using liquid nitrogen and then stored at −80 °C until further use.

### Sample identificaiton

To preserve the integrity of the RNA within the samples, the collected sandflies were first quickly identified by a magnifying lens at the subfamily (Phlebotominae) level using morphological characteristics listed below: (i) The adult head has large compound eyes and a pair of multiple antennae that are very long. Each antenna has 16 nodes: the base node is short, the second node is spherical, and the remaining 14 nodes are whip-shaped. And the mandible is very long, with 5 sections each, arranged on both sides of the beak and bent backward; (ii) a pair of wings on the chest is shaped like peach leaves. When resting, the wings and the chest form a "V" shape; and (iii) there are erect tufts or prone hairs on the back of the second to sixth sections of the back of the abdomen [24]. The species identification was later carried out after the processing and sequencing of sampling, and it is based on analyzing the consensus cytochrome C oxidase subunit I (COI) gene derived from the meta-transcriptomics sequencing results.

### RNA extraction, library construction, and sequencing

Sandfly specimens were grouped into 10 pools based on sampling location date, with each pool containing 50–100 sandflies. After being ground manually in the MEM medium, the homogenized samples were centrifuged at 12,000 rpm for 30 min at 4 °C to remove sandfly debris. Total RNA extraction and purification were carried out using the RNeasy Plus universal mini kit (QIAGEN, Germany) according to the manufacturer’s instructions. RNA sequencing library construction and ribosomal RNA depletion were performed using the Zymo-Seq RiboFree™ Total RNA Library Kit (Zymo Research, the United States). Paired-end (150 bp) sequencing of the 10 dual-indexed libraries was performed on a Novaseq platform at Novogene, China.

### Virus discovery and genome annotation

Low-quality reads and adaptor sequences were first removed from the raw sequencing data using bbmap (https://sourceforge.net/projects/bbmap/), and the resulting clean reads were then assembled *de novo* into contigs using Megahit (version 1.2.8) [25]. The assembled contigs were compared against the non-redundant protein (nr) database downloaded from GenBank using Diamond blastx (version 0.9.25) [26] with an *E*-value threshold of 1*E*-5. Potential viral contigs were obtained based on the taxonomic information of the blast hits and were then merged into longer genomes using the seqman program implemented in the Lasergene software package version 7.1 (DNAstar) [27]. Host contigs misidentified as virus genomes were removed by comparingthem against the non-redundant nucleotide database (nt). To identify potential novel virus species, we used a threshold of <90% amino acid identity for the RdRp (RNA-dependent RNA polymerase) protein (RNA viruses), DNA polymerase B protein (*Adintoviridae*), and nonstructural protein 1 (*Parvoviridae*). To avoid misassembly, reads were mapped back to the virus genome with Bowtie2 (version 2.3.5.1) [28] and inspected/corrected with Geneious Prime (version v.9.1.5) [29].

### Viral abundance estimation

Clean reads associated with ribosomal RNA were first removed by mapping against the ribosomal RNA database downloaded from the SILVA website (https://www.arb-silva.de/) using Bowtie2 [28]. The remaining unmapped reads were subsequently assigned to genomes of each viral species using the “end-to-end” mapping algorithm implemented in the Bowtie2 program. The abundance of each virus was subsequently determined as reads per million (RPM) using the formula “total mapped reads/total non-rRNA reads * 1,000,000”. In addition, false positives due to index hopping were removed at a threshold of 0.1% for each species present in the same sequencing lane.

### Identification and abundance estimation of bacteria and *Leishmania* parasites

Bacteria and eukaryotic microbes were firstly identified using MetaPhlAn2 [30]. Further species identification was carried out for *Rickettsia*, *Wolbachia*, *Leishmania*, and a novel member of the family Trypanosomatidae. Specifically, marker genes (groEL for *Rickettsia* and *Wolbachia,* hsp70, and beta-tubulin for *Leishmania*) were used to conduct similarity comparisons and phylogenetic analyses in order to determine which species or evolutionary group these microbes belong to. To estimate the relative abundance of each taxon in each library, the complete reference genome sequences of each genus were downloaded from GenBank and used as a mapping template for abundance estimation (RPM). To avoid bias in abundance estimation, highly conserved regions, such as rRNA genes, were removed from reference genome sequences before mapping analyses were carried out.

### Phylogenetic analysis

To determine the evolutionary history of the microbiome discovered here, we performed phylogenetic analyses based on protein and/or nucleic acid sequences of the conserved genes. For RNA viruses, RNA-dependent RNA polymerase (RdRp) of these viruses was aligned with those related viruses retrieved from GenBank using mafft [31]. Ambiguously aligned regions were subsequently removed using TrimAl [32]. Phylogenetic trees were then estimated by the maximum likelihood (ML) approach implemented in PhyML version 3.0 [33], employing the LG model of amino acid substitution and the Subtree Pruning and Regrafting (SPR) branch-swapping algorithm. An approximate likelihood ratio test (aLRT) with the Shimodaira-Hasegawa-like procedure was used to assess the support for individual nodes. For DNA viruses, DNA polymerase B (*Adintoviridae*) and NS1 protein (*Parvoviridae*) were used for phylogenetic analyses as described above. For bacteria and eukaryotes, nucleotide sequences of marker genes (groEL for *Rickettsia* and *Wolbachia,* hsp70, and beta-Tubulin for *Leishmania*) were used for phylogeny inference.

### Statistical analyses

For each library, the alpha diversity was estimated by the Shannon Wiener index at the species level using the *vegan* package version 2.5-6 implemented in R. The overall significance level was evaluated using ANOVA. Beta diversity was estimated based on the covariance matrix and evaluated using the Principal Component Analysis [34], and the overall significance level was evaluated using ADONIS. All the analyses were performed using R version 4.0.1 implemented in RStudio version 1.3.959 [35]. Graphs were plotted with the *pheatmap* and *ggplot2* packages [36].

## Results

### Sandfly meta-transcriptomics sequencing and species identification

**Figure 1.**
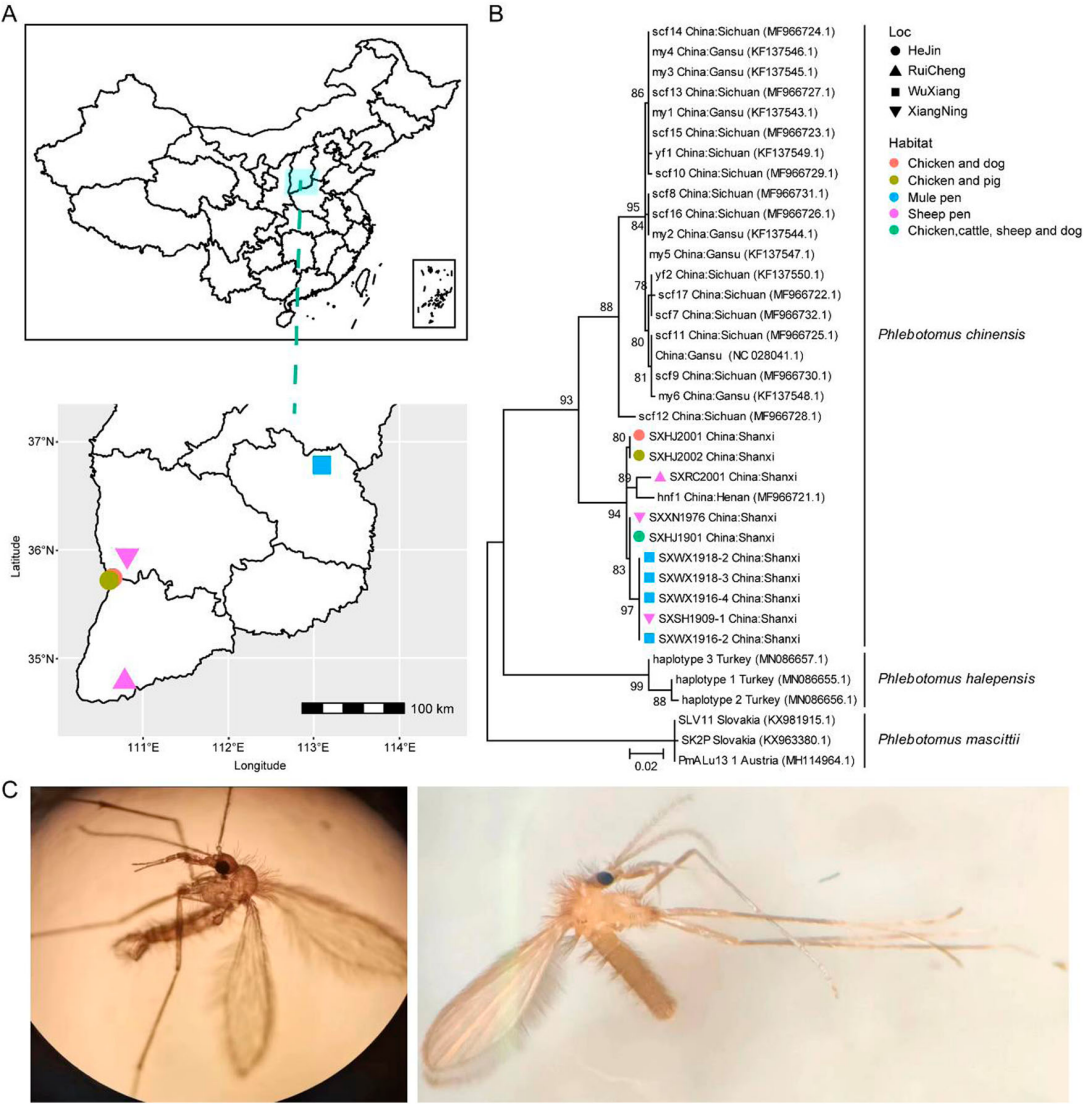
Sample locations and species identifications. (A) The locations of different sandfly pools sequenced in this study. (B) Species identification based on the cox1 gene. (C) The morphological features of sandflies collected from Shanxi province. Throughout the figure, we use different shapes to represent location information and different colours to indicate habitat information.

Meta-transcriptomics sequencing was performed on ten pools, containing 744 sandflies collected from four locations in Shanxi province of China (Figure 1(A), Table 1). It generated 50,495,976–75,003,498 reads per pool, which were subsequently *de novo* assembled to form 69,337–215,424 contigs (Table S1). Among these, contigs associated with cytochrome c oxidase subunit 1 mitochondrial gene (i.e. cox1 gene) were used to confirm the morphologically identified sandfly species, that is, *Phlebotomus chinensis*. Importantly, the cox1 sequences obtained from the ten sequencing pools were highly similar to each other, with 97.6-100% nucleotide identities. Phylogenetic analyses revealed that these sequences formed a single cluster nested within the diversity of *Ph. chinensis* and related to a single sample, that is, hnf1 (MF966721.1, 97.8 ∼ 98.1% nucleotide identity), sampled from the Henan province (Figure 1(B)), suggesting a relatively homogenous population across different sampling regions (Figure 1(C)). To investigate the sandfly diversity within each pool, we analyzed the proportion of minor variants (minimum coverage > 20, minimum variant frequency > 5%) based on reads mapped to the COI gene sequences. Nucleotide polymorphism was identified in 0% ∼ 1.83% of the sites for these pooled samples (Table S2), suggesting variation in the population’s genetic background, although the proportion of polymorphic sites was too low to imply the presence of another species (> 10% genetic differences). Therefore, *Ph. chinensis* was most likely to be the dominant sandfly species within these pools.

**Table 1.** Sample information for each of the sequencing pools.

Pool ID	Sampling site	Sampling time	Number of Sandflies	Breeding site	Longitude and latitude
SXHJ1901	HeJin	201906	50	Chicken, cattle, sheep, and dog	110.649437, 35.740849
SXHJ2001	HeJin	202007	50	Chicken and dog	110.649437, 35.740849
SXHJ2002	HeJin	202007	50	Chicken and pig	110.610682, 35.718501
SXRC2001	RuiCheng	202007	50	Sheep pen	110.790333, 34.78076
SXSH1909-1	XiangNing	201906	100	Sheep pen	110.846585, 35.970834
SXWX1916-2	WuXiang	201906	89	Mule pen	113.092729, 36.784861
SXWX1916-4	WuXiang	201906	88	Mule pen	113.092729, 36.784861
SXWX1918-2	WuXiang	201906	93	Mule pen	113.092729, 36.784861
SXWX1918-3	WuXiang	201906	93	Mule pen	113.092729, 36.784861
SXXN1976	XiangNing	201906	81	Sheep pen	110.846585, 35.970834

### Characterization of sandfly total microbiome

**Figure 2.**
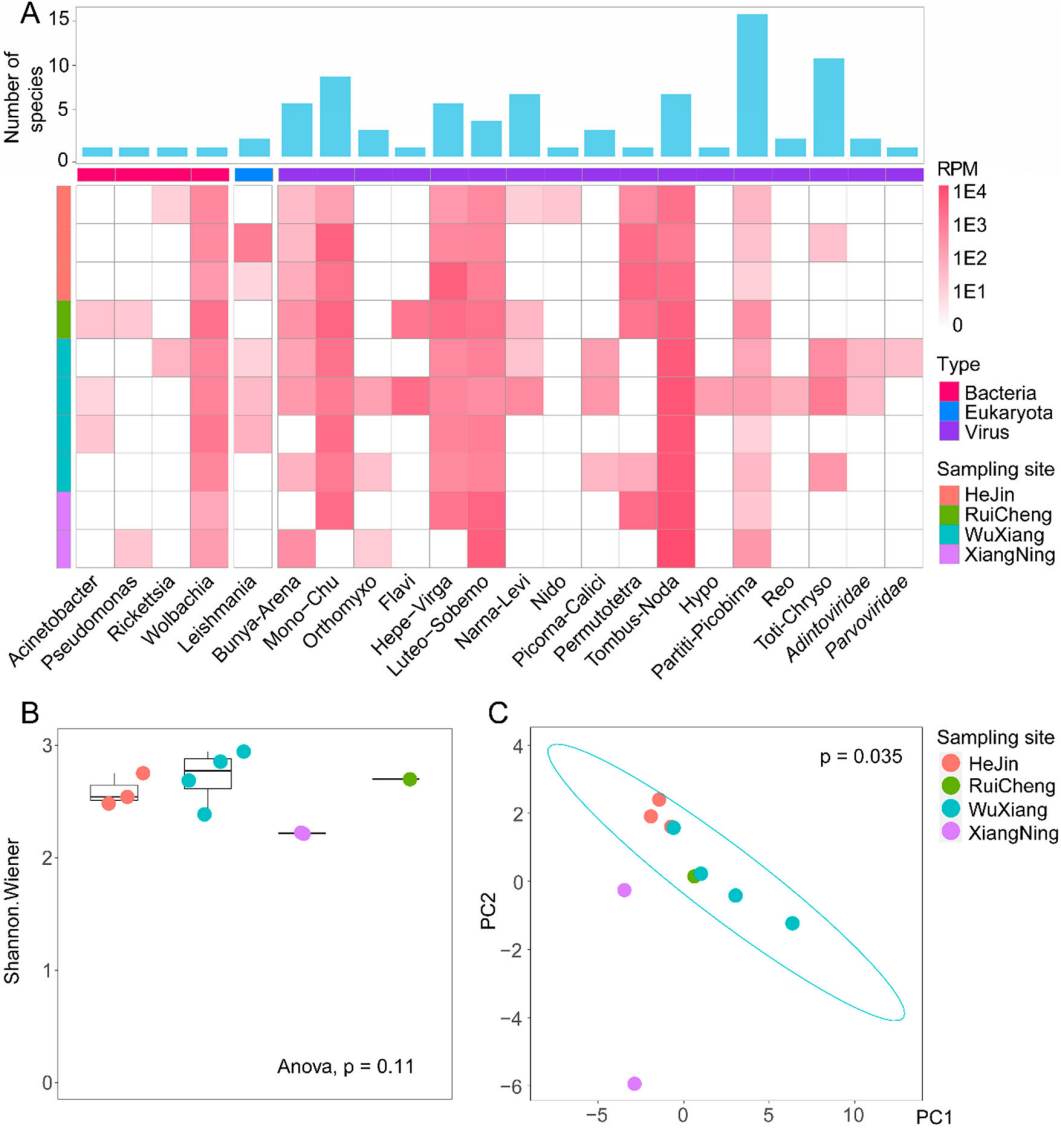
Total microbiome characterization of sand flies. (A) The abundance and species richness of major types of microbes in sandflies revealed by meta-transcriptomic sequencing. The heatmap shows the microbial abundance (measured by RPM), and the bar chart shows the number of species associated with each taxon. (B) The comparisons of alpha-diversity across the four sampling locations. (C) Principal component analysis (applying the covariance matrix) for microbial composition across the four sampling locations.

Our study revealed a number of actively replicating/transcribing viral, bacterial, and eukaryotic microbes within sandflies, with their abundance or intensity of biological activities measured by reads per million (RPM) (Figure 2(A)). Generally, the total microbiome is composed of 0.42-1.66% of total RNA, with RNA viruses being the most abundant, followed by bacteria, DNA viruses, and eukaryotic microbes (Figure 2(A)). The RNA viruses included a huge diversity of 15 viral super-groups [14]. On the other hand, members of DNA virus families, namely *Adintoviridae* and *Parvoviridae*, were also revealed from meta-transcriptomics sequencing, with moderate abundance levels (36.80–57.22 PRM). As for bacteria, four genera, namely *Acinetobacter*, *Pseudomonas*, *Rickettsia*, and *Wolbachia*, had higher abundance levels and genome coverage rates than other bacteria so they were revealed with great certainty by our RNA sequencing. Among these, *Wolbachia*, an endosymbiont found in many insect species [37], is present in all pools examined in this study, with relatively high abundance levels (i.e. 131–2712 RPM). For eukaryotic microbes, *Leishmania* was identified from five of the ten pools, and its abundance level varied from 10 to 1472 RPM. Other pools also contain *Leishmania*-associated reads, although they were below the threshold to be considered positive (i.e. 10 RPM). Overall, the alpha-diversity of microbes, including viruses, bacteria, and eukaryotic microbes, was quite even across all libraries (Figure 2(B)), and the microbial compositions could not be distinguished by locations (Figure 2(C)), suggesting a lack of geographic structure on microbial diversity in the four locations we investigated.

### The virome of *Ph. chinensis*

This is the first time that the total virome of sandfly is systematically characterized. More than 78 species of RNA viruses were discovered in this study, the majority of which (66/78) were new viruses based on the demarcation criteria of <90% amino acid identity at the replicates (Table S3). The Supergroup Partiti-picobirna (*n* = 16) contained the highest number of species, followed by Supergroups Toti-Chryso (*n* = 11), Mono-Chu (*n* = 9), and Narna-Levi (*n* = 7). In comparison, only three species of DNA viruses were identified, among which two belonged to the family *Adintoviridae* and one to *Parvoviridae*.

Among these, several viruses, namely, Shanxi sandfly flavivirus from Flavi, Shanxi sandfly virga-like virus 1–3, and Hubei macula-like virus 3 from Hepe-Virga, Shanxi sandfly sobemo-like virus from Luteo-Sobem, Shanxi sandfly rhabdovirus, and Shanxi sandfly mononega-like virus from Mono-Chu, Shanxi sandfly permutotetra-like virus from Permutotetra and Shanxi sandfly tombus-like virus 1–3 from Tombus-Noda had exceptionally high abundance levels (> 1000 RPM) and were found in more than half of the pools sequenced in this study (Table S3), suggesting high prevalence within the sandfly population examined here. Therefore, for new viruses with larger than 1000 RPM, host information was added to their naming because they are most likely to be associated with principal host *Ph. chinensis* rather than with food, parasites or commensal microbes within the host. On the other hand, for a number of viruses with low abundance and low pool prevalence rate, their corresponding host association remains unclear.

Within each defined supergroup or family, the majority of the viruses were clustered within the viral groups harboured by other insect or arthropod hosts, and some shared close phylogenetic relationship (> 90% amino acid identity) with viruses identified from mosquitoes (i.e. Zhee Mosquito virus, Menghai rhabdovirus, amongst others), aphids (Aphis citricidus meson-like virus), spiders (Hubei macula-like virus 3 and Guiyang nodavirus 2), and amongst others (Figure S1–S4). Nevertheless, a few viruses with low abundance levels were bona fide fungal pathogens, including Fusarium graminearum hypovirus 2 (Hypo), Alternaria alternata partitivirus 1 (Partiti-Picobirna), Botrytis cinerea partitivirus 2 (Partiti-Picobirna), and Fusarium poae dsRNA virus 3 (Toti-Chryso). Furthermore, several newly identified species (e.g. Shanxi partiti-like virus 4, 5, 6, 9, 10, and 12, and amongst others) under Supergroup Partiti-Picobirna were clustered within the diversity associated with viruses in basal eukaryotes (Figure S3), suggesting their potential association with the non-sandfly microbial host.

**Figure 3.**
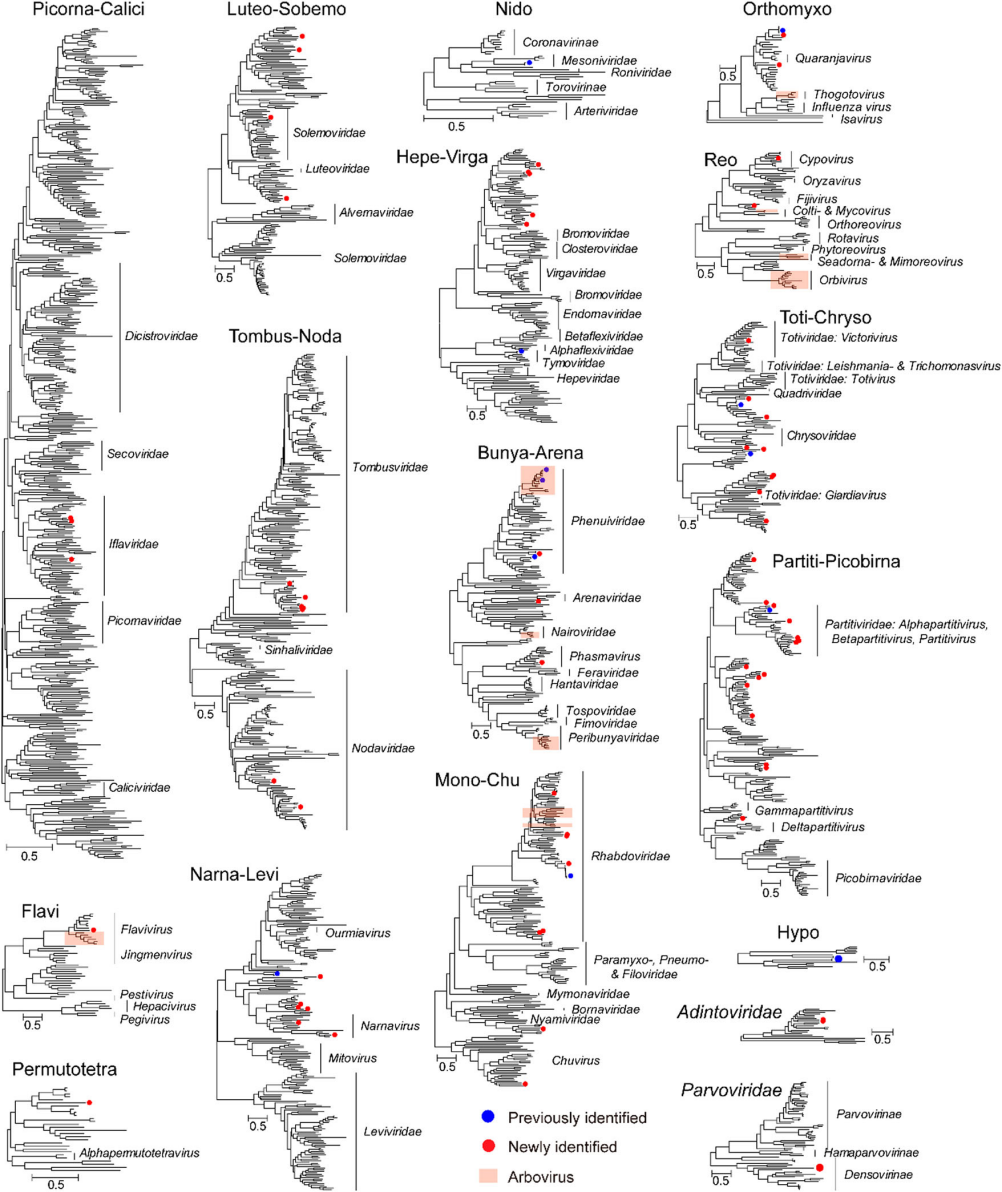
The diversity and evolutionary relationship of viruses identified in this study. 17 phylogenetic trees are shown, among which 15 are based on RNA-dependent RNA polymerase (RdRp) of major RNA virus supergroups, one is on DNA polymerase B protein of *Adintoviridae*, and one on NS1 protein of *Parvoviridae*. Within each phylogeny, existing virus species are marked with blue circles, while new virus species are marked with red circles. In the phylogeny, we use orange shaded boxes to mark the diversity associated with an arbovirus. The names of the families or genera within each clade are shown to the right of each phylogeny.

Among the discovered viruses, we further searched for viruses that can infect humans or other mammalian hosts, namely, arthropod-borne viruses or arbovirus, based on the phylogenetic relationship with the established arthropod-borne virus genera or groups, including genera *Flavivirus*, *Alphavirus*, *Phlebovirus*, *Orbivirus*, amongst others (Figure 3). Only two viruses fell within the arthropod-borne virus (arbovirus) categories, namely wuxiang virus and hedi virus, both were related to genera *Phlebovirus* under the Order *Bunyavirales*, and had been isolated in previous studies using mammalian cell lines [10–13]. Interestingly, a novel member of the genus *Flavivirus* was also identified in this study, with greater than 1000 RPM abundance levels in two the pools (Figure S5, Table S1). Nevertheless, it did not cluster with mosquito-borne or tick-borne virus groups, which were known arboviruses but was presented as a divergent lineage clustered with a classic insect-specific flavivirus, which included a newly identified flavivirus associated with the Japanese horse fly (44.78% amino acid identity) [38] as well as viruses associated with mosquitoes, namely, Quang binh virus, Cell fusing agent virus, Aedes flavivirus, amongst others, and, therefore, further expanded the diversity and host range of classic insect-specific flaviviruses.

### The bacteria composition within *Ph. chinensis*

**Figure 4.**
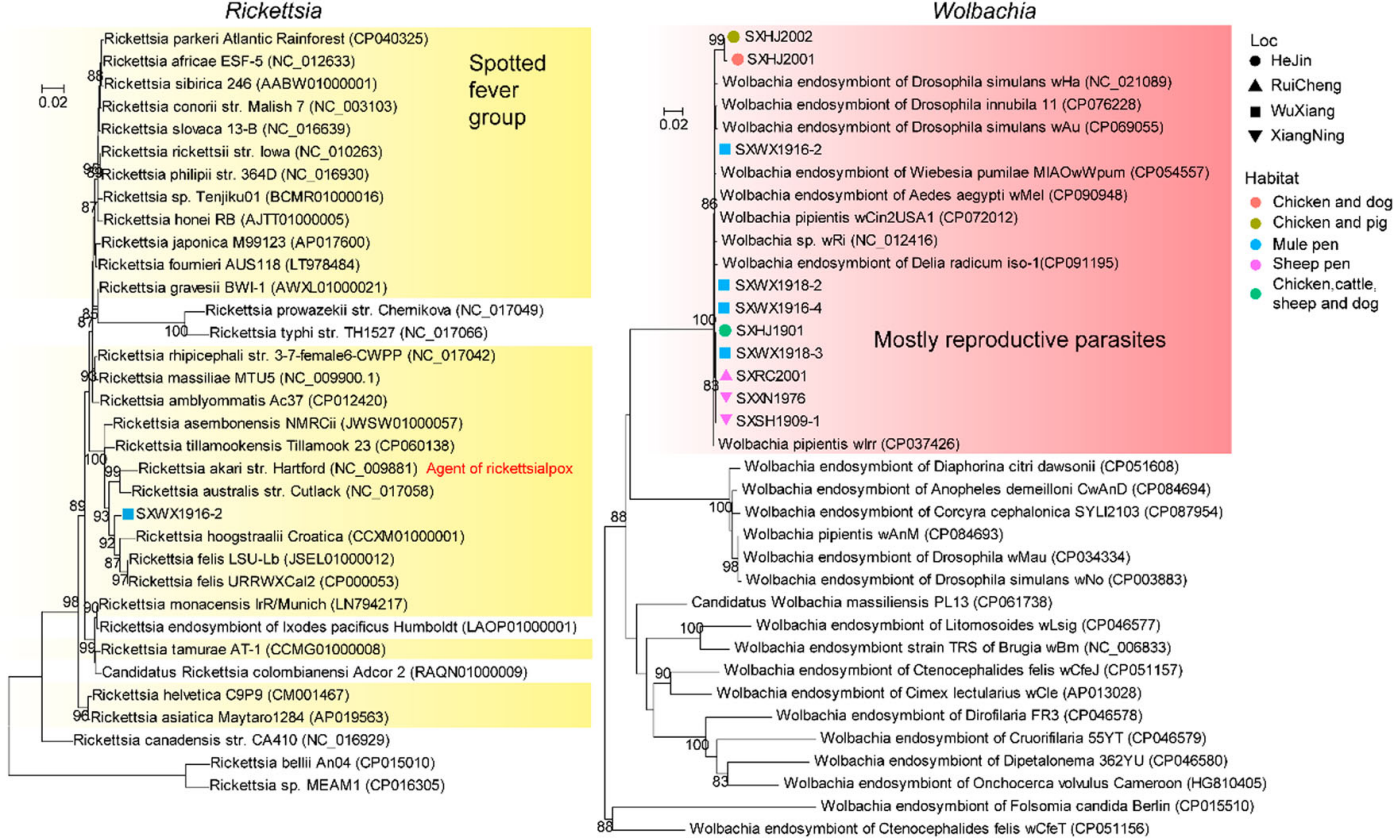
Identification of *Rickettsia* and *Wolbachia* bacterium at the species level. Phylogenetic trees based on the groEl gene showing the position of newly identified bacteria within the diversity of genera Rickettsia (left) and *Wolbachia* (right). We use different shapes to indicate location information and different colours to indicate habitat information. Specific groups such as the spotted fever group and mostly reproductive parasites are highlighted with a shaded box on the Rickettsia and *Wolbachia* trees, respectively.

We have identified four bacteria of relatively high abundance levels from meta-transcriptomics: *Acinetobacter*, *Pseudomonas*, *Rickettsia*, and *Wolbachia*. The *Rickettsia* identified in this study belonged to the well-established Spotted fever group (Figure 4(A)), and it was most closely related to *Rickettsia felis* (97.37% identity at GroEL gene), the causative agent of flea-borne spotted fever in humans [39]. Given the divergence level at the GroEL gene, the *Rickettsia* discovered here to merit the assignment of a new species. On the other hand, *Wolbachia* was detected in all pools examined in this study and was genetically quite closely related to a group containing most of the reproductive parasites associated with insect hosts, including *w*Mel, *w*Au, *w*Ri, amongst others, although it is still unclear based on current phylogeny that how many different strains were present in the pooled samples (Figure 4(B)). For both newly identified *Rickettsia* and *Wolbachia* bacteria strains, transcriptomics profiling revealed the expression of genes with diverse functions (Figure S6), suggesting that these were biologically active microbes within the host.

### Dominant eukaryotic microbes identified from *Ph. chinensis*

**Figure 5.**
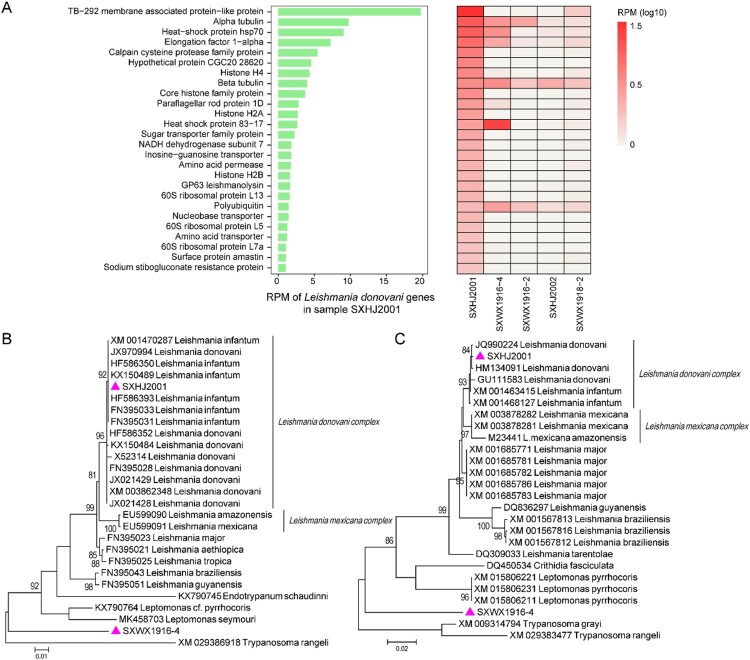
Transcriptomic profiles and identification of eukaryotic microbes. (A) Transcriptomic profiles of Leishmania in the pool SXHJ2001 (left) and the other positive pools (right). Phylogenetic position of Leishmania and a newly identified member of the family Trypanosomatidae based on (B) hsp70 and beta-tubulin genes (C). Throughout the figure, purple triangles represent the strains identified in this study.

Our data revealed at least two species of eukaryotic microbes. One was *Leishmania*, an intra-cellular parasite associated with visceral leishmaniasis in humans. It was detected in at least five of the ten pools examined in this study, each with slightly different transcriptomic profiles and abundance levels (Figure 5(A)). Phylogenetic analyses based on two of the most abundant genes, the heat shock protein 70 (hsp70) gene and beta-tubulin gene, revealed that it belonged to the *Leishmania donovani* complex (Figure 5(B,C)). However, it was unclear based on current data to which species, *Leishmania donovani* or *Leishmania infantum*, did it belong. In addition to *Leishmania*, we were also able to identify a divergent new member of the family Trypanosomatidae. While the top blast hits for this organism were *Leptomonas pyrrhocoris* (91.70% nucleotide identity) and *Leishmania mexicana* (92.15%) respectively based on hsp70 and beta-tubulin genes, phylogenetic analyses placed this newly identified microbe as orphan lineage sister to the entire subfamily Leishmaniinae, suggesting a highly distinctive organism.

### Epidemiological features of arthropod-borne pathogens

**Figure 6.**
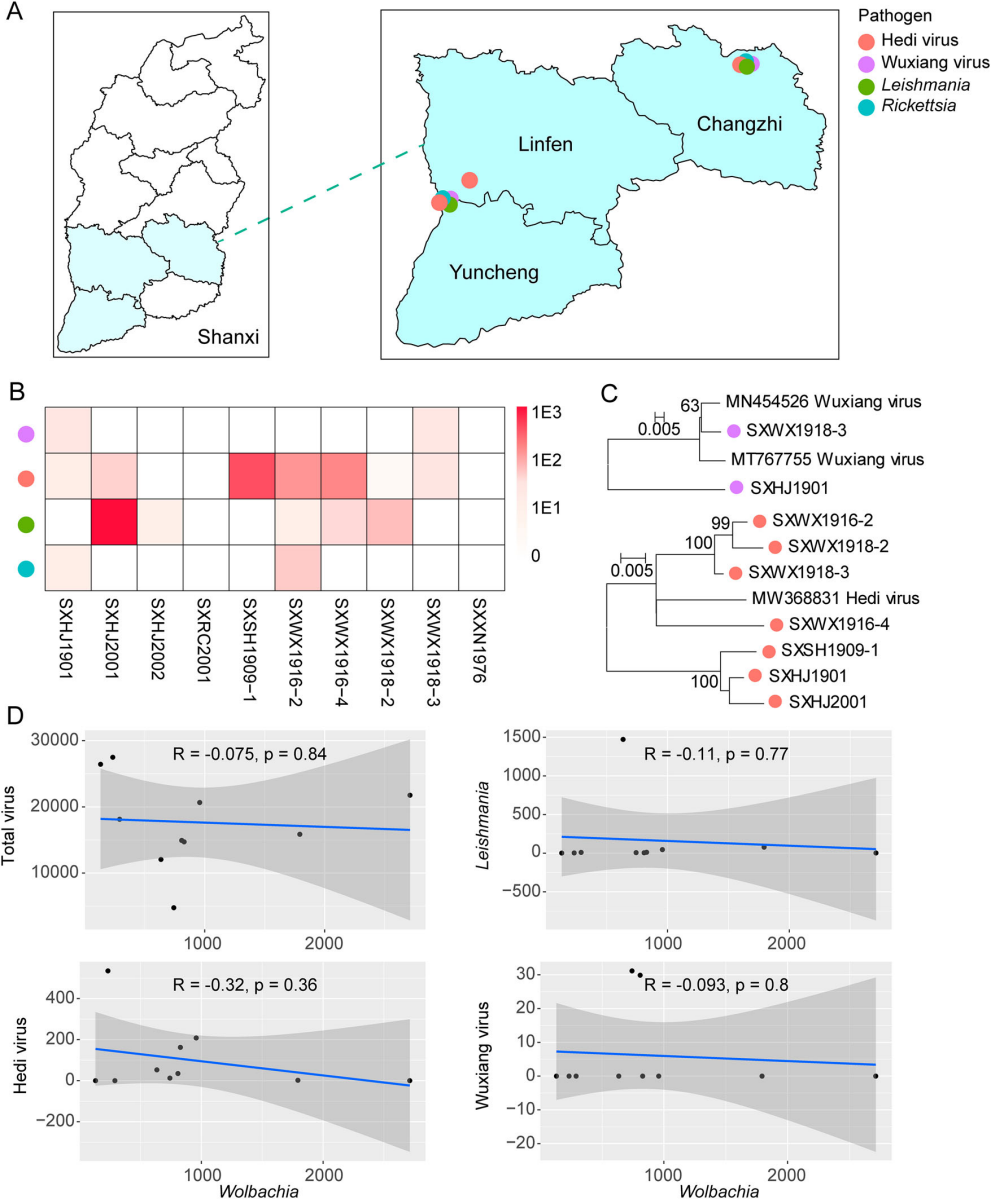
Epidemiological features of pathogens found in sand flies. (A) Distribution of arthropod-borne pathogens found in sand flies. (B) Phylogenetic analysis of hedi virus and wuxiang virus. (C) Abundance (measured in RPM) of each of the four pathogens across ten pools. (D) Correlation of abundance level between *Wolbachia* and the total virome, hedi virus, wuxiang virus, and Leishmania.

For epidemiological features, we focused on microbes that may cause infection in humans and other mammalian animals, which included hedi virus, wuxiang virus, *Rickettsia* sp., and a member of the *Leishmania donovani* complex. Among these, hedi virus was quite prevalent and distributed in Changzhi, Linfen, and Yuncheng, whereas the other three pathogens were distributed in Changzhi and Yuncheng (Figure 6(A)). Specifically, the pool prevalence rates are 7/10, 2/10, 2/10, and 5/10, respectively, for hedi virus, wuxiang virus, *Rickettsia,* and *Leishmania* (Figure 6(B)). And the pathogens made up an average of 0.028% of total non-ribosomal RNA reads within sandflies, while the total microbiome was likely greater than 1.8%. And the abundance percentage was 0.0081%, 0.00060%, and 0.019%, respectively for viral, bacteria, and eukaryotic pathogens. Furthermore, extensive intra-specific diversity was observed for both hedi and wuxiang viruses (Figure 6(C)), suggesting local endemic of these viruses and further implying a potentially longer evolutionary history and greater geographical span than currently depicted. Finally, we also examined whether *Wolbachia* has any protective effect against these pathogens since it is experimentally proved that infection of *Wolbachia* can prevent the replication of arboviruses replications (e.g. Dengue virus) in mosquitoes [40]. We tested the correlation of abundance between *Wolbachia* and the total virome, hedi virus, wuxiang virus, and *Leishmania*, and none resulted in a significant and strong relationship (Figure 6(D)), suggesting no observable effect of naturally infected *Wolbachia* strain in reducing the abundance of arthropod-borne pathogens within sandflies.

## Discussion

Our study reveals that the sandfly is an important but neglected vector that harbours multiple types of infectious agents relevant to human health. Indeed, meta-transcriptomics sequencing has demonstrated with compelling molecular evidence that the presence and active biological activities of four pathogens or potential pathogens, including two viruses, one bacterium, and one eukaryotic microbe in *Ph. chinensis* sandfly samples, and among them, two (i.e. hedi virus and *Leishmania*) had high prevalence and abundance in the region investigated. In addition to our findings, another study based on 16S metagenomic sequencing of sandflies collected from Henan, a neighbouring province of Shanxi, has also identified bacteria close related to *Rickettsia bellii* [8], suggesting the presence of other sandfly-borne pathogens in northern China. Collectively, the evidence so far indicates high disease risks associated with *Ph. chinensis* in Northern China. Furthermore, the diversity and overall abundance of the pathogen in sandflies estimated in this study is much higher than another important insect vectors, mosquitoes, which mainly harboured viral pathogens with 0-0.0059% abundance levels based on the same meta-transcriptomics sequencing [20,22,41], although those studies on mosquito microbiome were performed at different regions.

The disease caused by *Leishmania* has been frequently reported in Shanxi province, which recorded 21.7% of the total mountain-type zoonotic visceral leishmaniasis cases between 2015 and–2019 in China, and, therefore, it is viewed as one of the hotspot regions for visceral leishmaniasis [42]. Furthermore, previous studies based on real-time quantitative PCR suggested that the average prevalence of *Leishmania* was 1.98% in female *Ph. chinensis*, indicating that the parasites were endemic within *Ph. chinensis* population [43]. While the public health significance of *Leishmania* parasites was well-recognized, the impact of other pathogens, namely, Rickettsia, hedi virus, and wuxiang virus discovered in this study, is unclear. One possibility is that these pathogens may cause sporadic self-limiting disease with non-specific symptoms, such as fever, rash, nausea, etc., which was reported in some infections caused by phleboviruses [44] and rickettsia [45]. Without active surveillance, patients with these types of diseases are often not subject to hospitalization for further pathogen screening and identification. Alternatively, these pathogens might be harboured by non-human mammalian hosts, since the sandflies are mainly feeding on domestic and wild-life animals instead of humans which had much less exposure. Regardless, the potential role of these pathogens in causing emerging infectious diseases in humans cannot be overlooked, because (i) they share a close relationship with human pathogens, (ii) hedi and wuxiang viruses are isolated and grown in mammalian cell lines, and (iii) serological data reveal the exposure of local human populations to wuxiang virus [46]. Therefore, it is important to actively monitor these potential pathogens and at the same time fully characterize their disease-causing capability such that future outbreaks can be prevented.

In addition to those causing human disease, our study shows the entire virome of *Ph. chinensis* sandflies. Like what has been revealed in other arthropod species, such as mosquitoes [20,22], ticks [47], flies [48], bees [49] and termites [50], the virome of sandflies is characterized by an enormous diversity that expands multiple viral super-groups and with a great abundance level that makes up an average of 1.67% total non-ribosomal RNA. While the majority of the virome is likely associated with the principal host (i.e. *Ph. chinensis*) based on the abundance levels, pool prevalence rate, and/or their phylogenetic positions, only a very small fraction is relevant for human infections (i.e. hedi virus and wuxiang virus), which is similar to the observations made in other blood-sucking arthropods, such as mosquitoes [20,22,41], ticks [47], midges [51], and batflies [48]. Also being revealed as part of the virome are a large diversity of viruses associated with parasites, endosymbionts, or foods. While it is unlikely to identify the host association for all viruses discovered here, these viruses were easily distinguished from the principal virome with characteristics, such as a low abundance level and a close relationship with non-host organisms [52].

Also of interest is the observation that the *Ph. chinensis* sandflies virome revealed here has substantial diversity overlap with the mosquito virome. Indeed, some viruses discovered here belonged to well-established mosquito virus species, such as the Zhee Mosquito virus [15], Hubei mosquito virus 3 [14], and Wuhan Mosquito Virus 7 [15], etc. It is possible that this overlap may be due to a common niche (i.e. feeding on the same animals) shared by the two distinguished vector species. Alternatively, the pooled sample may have been contaminated by a mosquito-associated tissue (e.g. a broken limb or the inner content of a smashed body). Nevertheless, it is unlikely that a non-sandfly insect (e.g. a mosquito) could be mistakenly placed in the pool given that each sandfly was independently identified and sorted.

Our study also revealed the diversity of bacteria and eukaryotic microbes from the *Ph. chinensis* sandfly populations in Shanxi province, mainly through their non-ribosomal transcriptomic profiles. Nevertheless, the bacteriome obtained here is strikingly different from those described in a previous survey based on the 16S sequencing, and the latter study was carried out on the same sandfly species sampled in the neighbouring provinces [8]. On the one hand, there are huge differences in the bacterial taxonomy richness, with the 16S-based approach revealing more diversity than the meta-transcriptomics-based approach, although it is generally expected that non-ribosomal tools, such as MetaPhlAn, are often overly conservative in terms of the abundance required for positive calling such that only those with higher abundance levels are revealed [53,54]. On the other hand, the most dominant bacteria groups differ between the two studies: in our study, the endosymbiont *Wolbachia* was detected in all libraries whose abundance levels are orders of magnitude higher than the other bacterial taxa, whereas the 16S-based approach reveals *Pseudomonas*, *Enterobacteriaceae*, and *Coxiellaceae* as the dominant taxa. While it is possible that such discrepancies are caused by sampling locations or subpopulations of sandflies, it could also be due to differences in the target nucleic acids, namely, DNA or RNA, sequenced in these two studies. The latter is more likely given that RNA sequencing is prone to reflect active microbial populations and hence results in much higher abundance levels for endosymbionts than those estimated by DNA sequencing [55].

Our study reveals that *Wolbachia*, a reproductive parasite affecting a wide range of invertebrate hosts [37], is present at a high pool prevalence rate and great abundance level within the *Ph. chinensis* sandfly populations in Shanxi. The organism has gained increasing interest in its potential use as a vector-borne disease control agent, owing to its capability to reduce the vector population by a mechanism of cytoplasmic incompatibility [56] as well as reported propensity to reduce the replication of arboviruses such as dengue virus and zika virus in mosquitoes [40,57]. Nevertheless, our data did not reveal a negative relationship between *Wolbachia* infection and the presence and abundance of arthropod-borne pathogens, namely, hedi virus, wuxiang virus, and *Leishmania*. One explanation is that not all *Wolbachia* strain is protectively against virus replications, as has been demonstrated based on a variety of *Wolbachia* strains in *Drosophila* [58,59]. Alternatively, it is also likely that the virus-blocking effect may be stronger under experimental conditions where the virus is transinfected into new host species, whereas no such effect is observed under natural conditions [21]. Indeed, the co-adaptation of the host, virus, and bacterial symbionts has been shaped over long time periods so that the interaction among them is likely to be much more complex than a simply blocking effect [60].

Our study presents an efficient yet comprehensive “total microbiome” approach for pathogen discovery in disease vectors such as sandflies. With a single meta-transcriptomics sequencing, it carries out a thorough investigation of viral, bacterial, and eukaryotic microbes under active biological processes within vectors, with further information from genome/transcriptome comparisons, and abundance estimation as well as phylogenetic analyses to identify potential pathogens and evaluate their threat to human health. This is especially relevant for sandflies where viral, bacterial, and eukaryotic pathogens were simultaneously present in the same region. Therefore, using the “total microbiome” approach it will prevent repeated efforts to discover different types of pathogens one at a time and provide the much-needed speed to detect emerging pathogens from a large number of vector samples.

Finally, our study had several limitations: first, the sampling scope is limited in terms of sandfly species and geographic regions covered, and a more systematic meta-transcriptomics survey is needed to fully capture the microbial diversity within different sandfly species and across a broader geographic range in China. Furthermore, the host association remains unclear for a number of low abundance level microbes with distinguished phylogenetic positions, and it is highly likely that they might be associated with food/symbionts instead of the sandflies. Finally, the potential role of the newly identified *Rickettsia* in human infections was only assessed with sequence data, which remains to be confirmed with data from tissue culture, animal experiments, and/or serological surveys. Nevertheless, despite the limitations, our study presents the first effort to characterize the total microbiome in sandflies, and such applications can be extended to other vector species, allowing more efficient discovery and characterization of potential pathogens in vectors.

## Supplementary Material

Supplemental Material

## Data Availability

All sequencing reads have been deposited in the SRA databases under the project accession PRJNA855979 and PRJNA855531. Relevant virus genome sequences and mitochondrial sequence data are available at the figshare website under the link: https://figshare.com/articles/dataset/virus_anno_fas/20224635.
