# Supplementary material for: Total RNA sequencing of Phlebotomus chinensis sandflies in China revealed viral, bacterial, and eukaryotic microbes potentially pathogenic to humans
Source: Emerg Microbes Infect. 2022 Aug 31;11(1):2080–92. doi: 10.1080/22221751.2022.2109516 (PMC9448391; doi:10.1080/22221751.2022.2109516)
Supplement: Supplemental Material [file TEMI_A_2109516_SM3117.zip › supplementary_materials.pdf]

Supporting information

Total RNA sequencing of sandfly, a neglected vector in China, simultaneous revealed viral, bacterial, and eukaryotic microbes that are potentially pathogenic to humans

Jing Wang [a, *], Qin-yu Gou [a, *], Geng-yan Luo [a, *], Xin Hou [a], Guodong Liang [b], Mang Shi [a, #]

[a] *The Center for Infection & Immunity Study, School of Medicine, Shenzhen campus of Sun Yat-sen University, Shenzhen 518107, China;*

[b] *State Key Laboratory of Infectious Disease Prevention and Control, National Institute for Viral Disease Control and Prevention, Chinese Center for Disease Control and Prevention, Beijing 102206, China;*

[*] These authors contributed equally to this work

[#] Co-corresponding authors:

Guodong Liang; gdliang@hotmail.com; State Key Laboratory of Infectious Disease Prevention and Control, National Institute for Viral Disease Control and Prevention, Chinese Center for Disease Control and Prevention, Beijing 102206, China

Mang Shi; shim23@mail.sysu.edu.cn; The Center for Infection & Immunity Study, School of Medicine, Shenzhen campus of Sun Yat-sen University, Shenzhen, China

Table S1

Table S1 The information of sequencing library of sandflies

Library	Species	Sampling site	Total reads	No-rRNA reads	Number of contigs
SXHJ1901	*Phlebotomus chinensis*	HeJin	55050938	41501909	172546
SXHJ2001	*Phlebotomus chinensis*	HeJin	62452978	43951704	154090
SXHJ2002	*Phlebotomus chinensis*	HeJin	61049490	41776406	131188
SXRC2001	*Phlebotomus chinensis*	RuiCheng	75003498	50590112	215424
SXSH1909-1	*Phlebotomus chinensis*	XiangNing	57759566	21927447	69337
SXWX1916-2	*Phlebotomus chinensis*	WuXiang	50623672	28345570	166024
SXWX1916-4	*Phlebotomus chinensis*	WuXiang	56329460	32703489	177714
SXWX1918-2	*Phlebotomus chinensis*	WuXiang	60049808	44607079	115841
SXWX1918-3	*Phlebotomus chinensis*	WuXiang	62331984	48745147	138738
SXXN1976	*Phlebotomus chinensis*	XiangNing	50495976	20412831	85264

Table S2 and S3

Table S2 Minor nucleotide variants of *Ph. chinensis* COI gene in each of the sequenced pools

Table S3 The information of viruses identified in this study

Figure S1

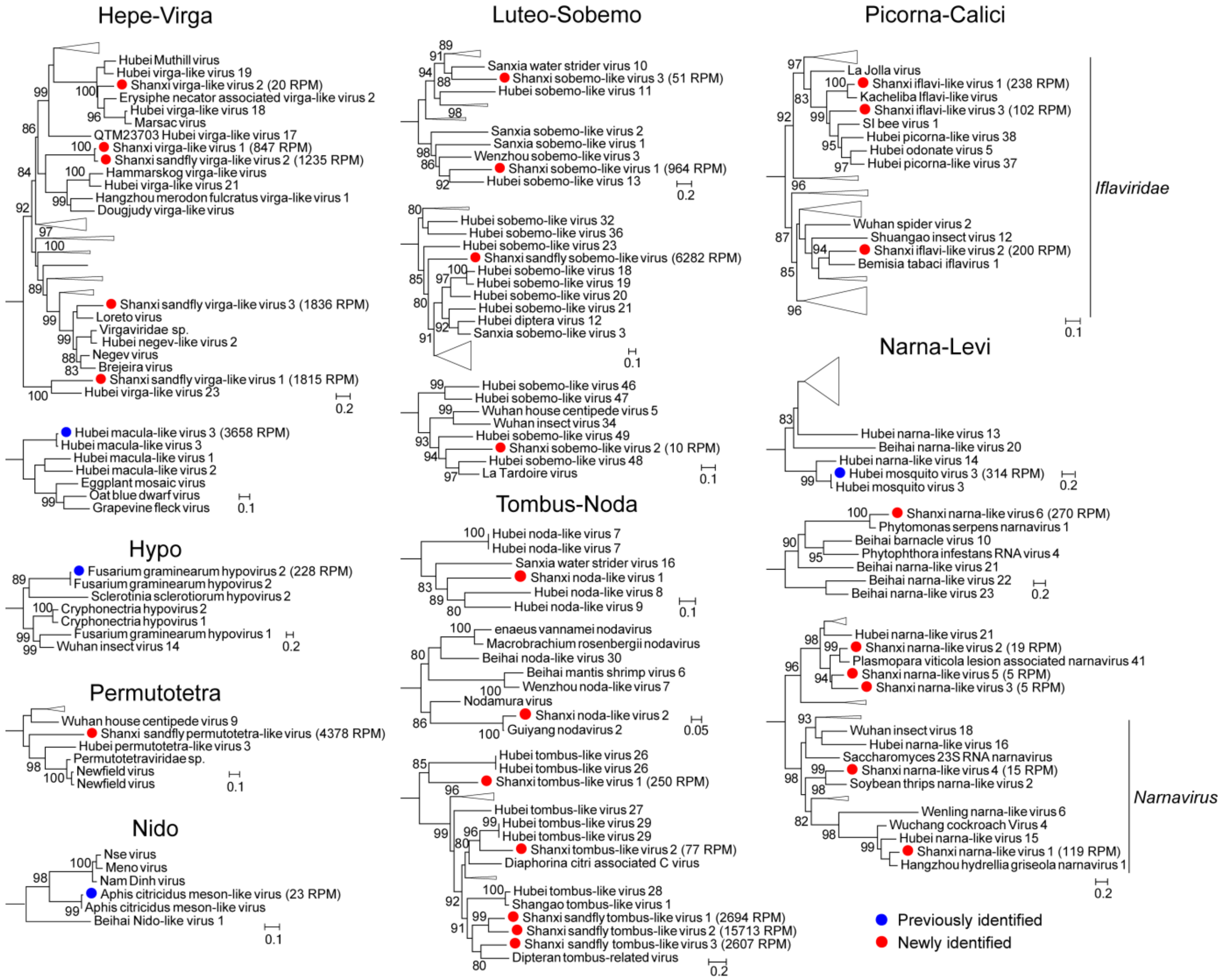

Fig. S1 Phylogenetic trees of Hepe-Virga, Hypo, Permutotetra, Nido, Luteo-Sobemo, Tombus-Noda, Picorna-Calici, and Narna-Levi groups (i.e. positive-sense RNA viruses). Within each phylogeny, red circles represent novel virus species, while blue circles represent viruses identified previously. The names of the families or genera within some clades are shown to the right of the phylogeny, while the abundance information (in parentheses) is provided after the names of the viruses identified here. Each scale bar indicates specific amino acid substitution per site.

Figure S2

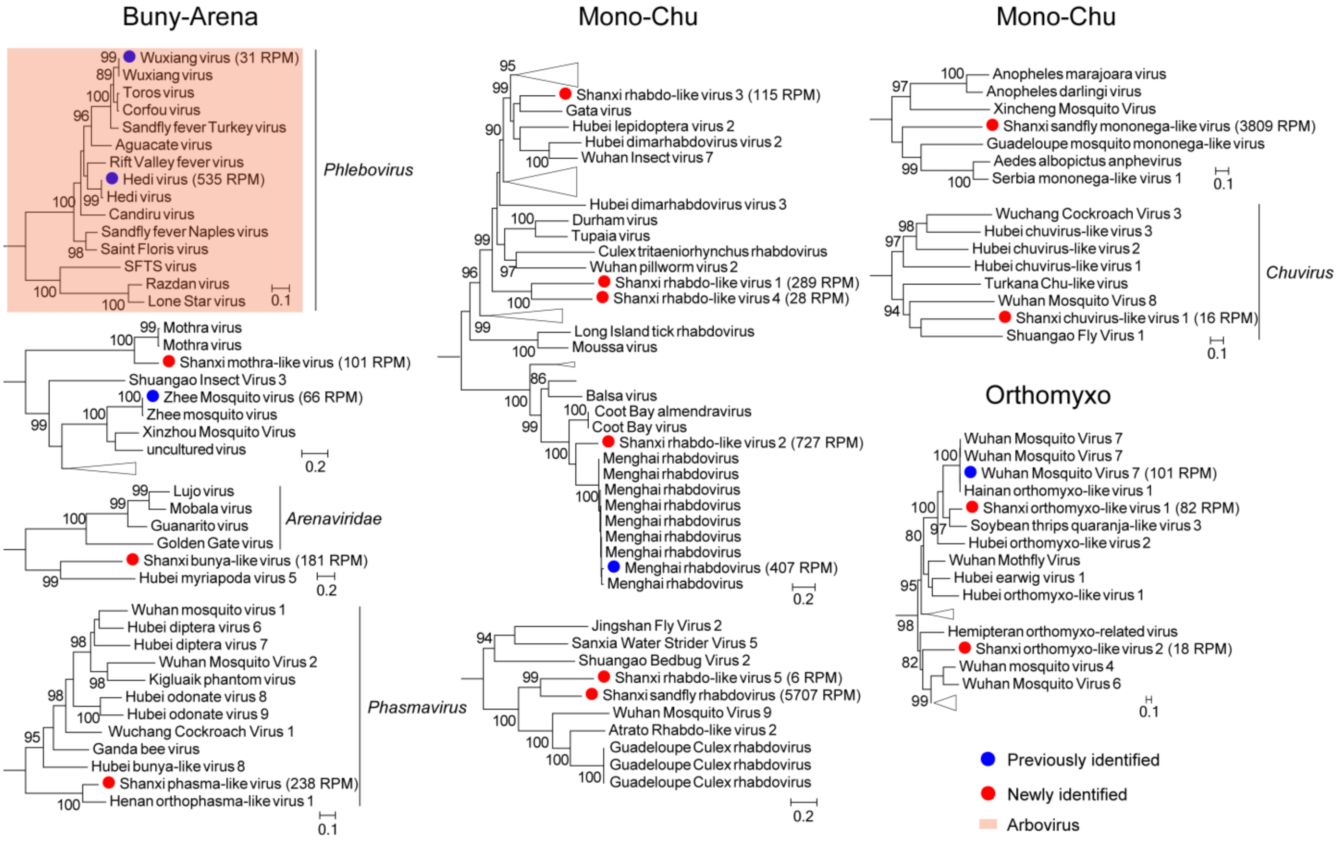

Figure S2. Phylogenetic trees of Buny-Arena, mono-Chu, orthomyxo groups (i.e. negative-sense RNA viruses)

Within each phylogeny, red circles represent novel virus species, while blue circles represent viruses identified previously. Shaded area represents arbovirus. The names of the families or genera within some clades are shown to the right of the phylogeny, while the abundance information (in parentheses) is provided after the names of the viruses identified here. Each scale bar indicates specific amino acid substitution per site.

Figure S3

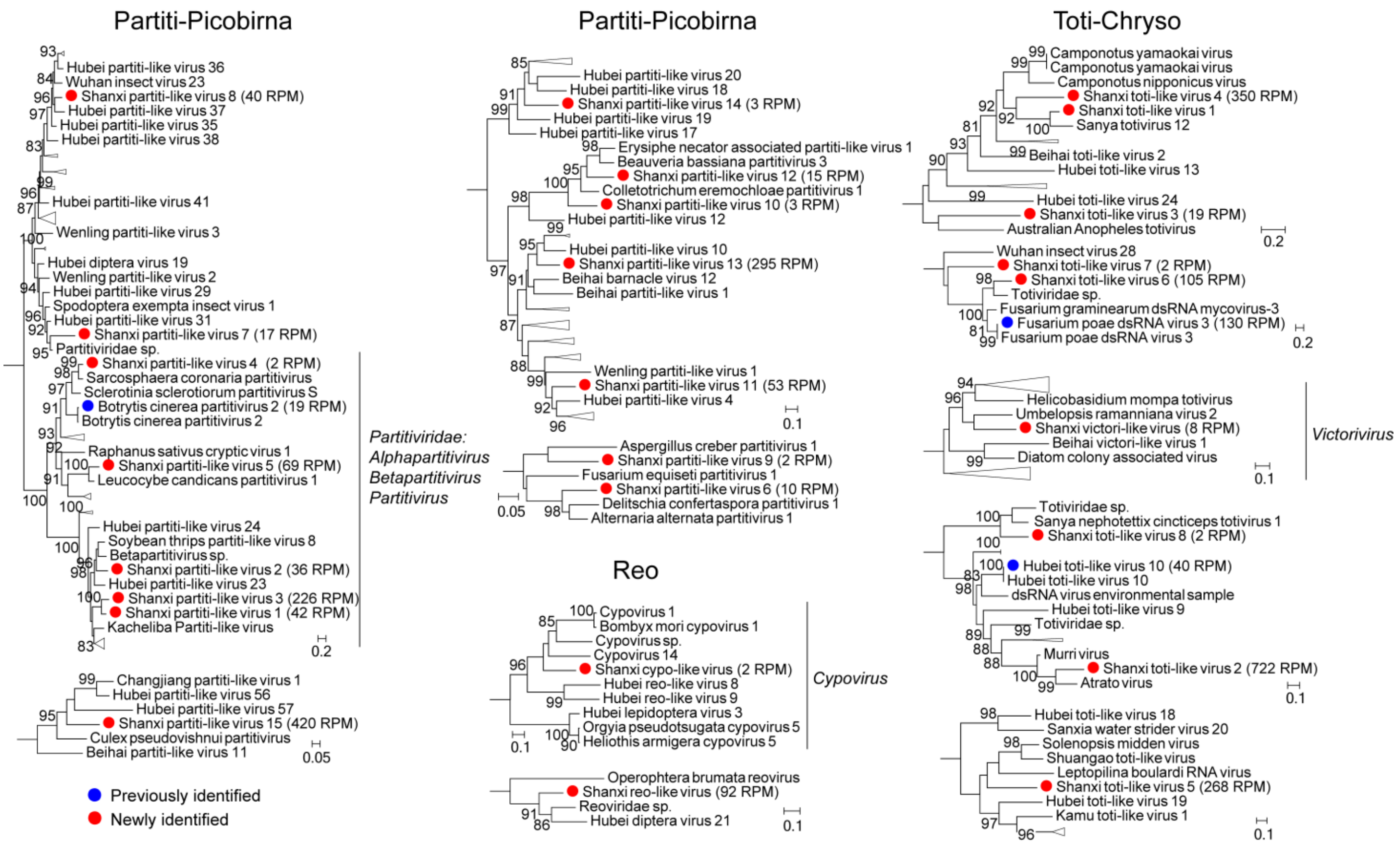

Fig. S3 Phylogenetic trees of Partiti-Picobirna, Reo, and Toti-Chryuso groups (i.e. double-stranded RNA viruses)

Within each phylogeny, red circles represent novel virus species, while blue circles represent viruses identified previously. The names of the families or genera within some clades are shown to the right of the phylogeny, while the abundance information (in parentheses) is provided after the names of the viruses identified here. Each scale bar indicates specific amino acid substitution per site.

Figure S4

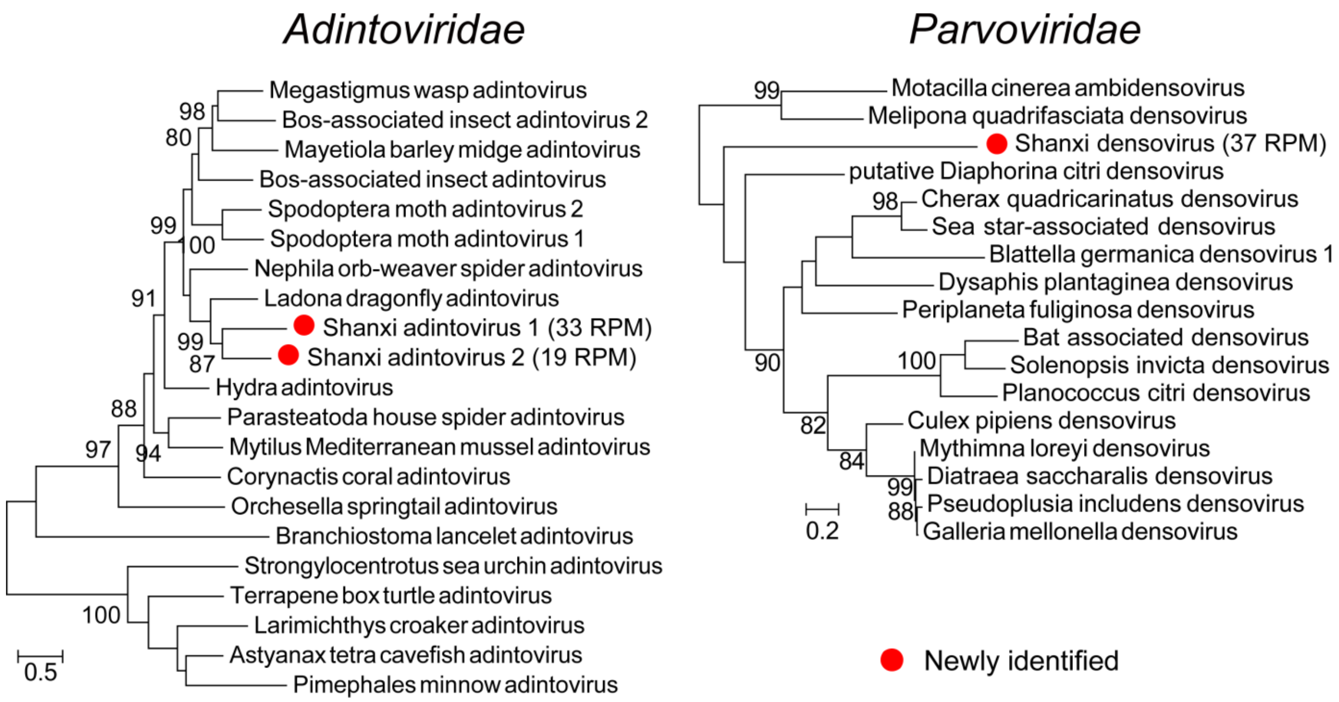

Fig. S4 Phylogenetic trees of *Adintoviridae* and *Parvoviridae*

Within each phylogeny, red circles represent novel virus species, while the abundance information (in parentheses) is provided after the names of the viruses identified here. Each scale bar indicates specific amino acid substitution per site.

Figure S5

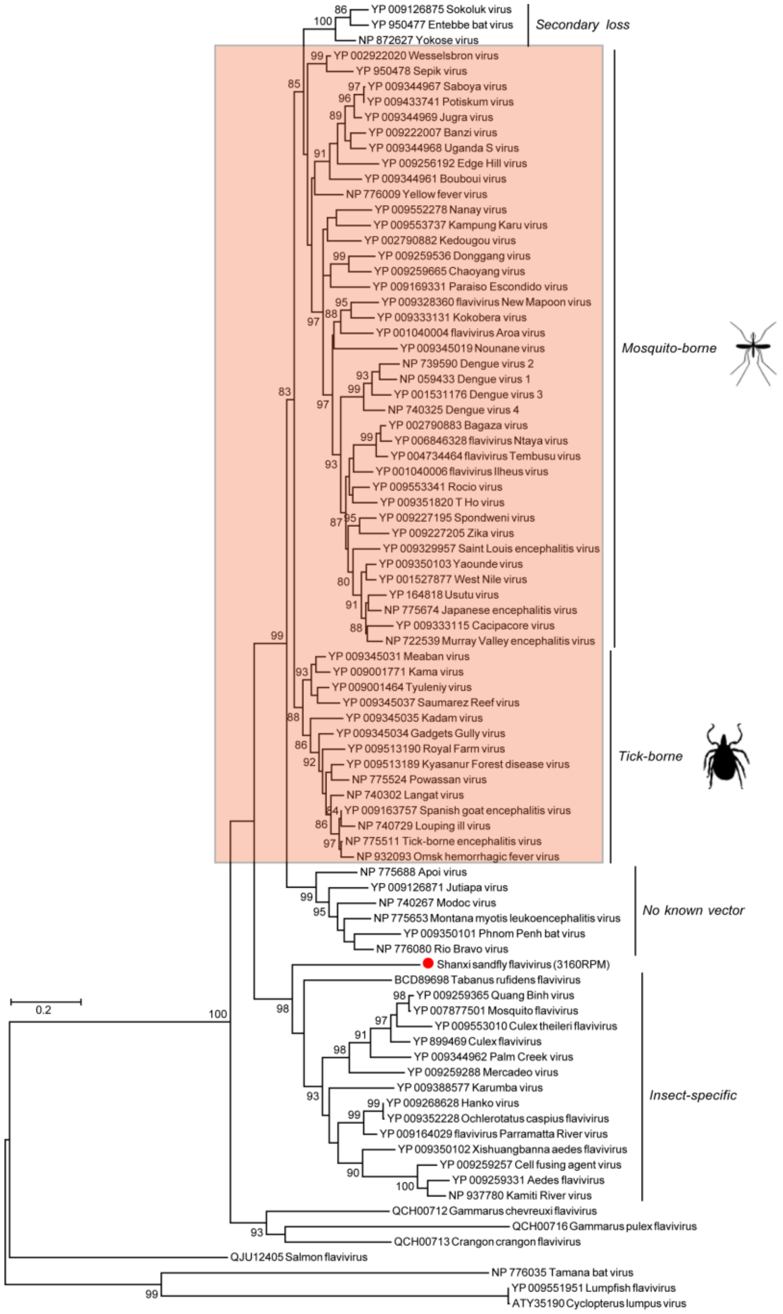

Fig. S5 Phylogenetic tree of *Flavivirus*. Within the tree, red circle represents novel virus species and shaded area represents arbovirus. The host of each clade are shown to the right of the phylogeny. The scale bar indicates 0.2 amino acid substitution per site.

Figure S6

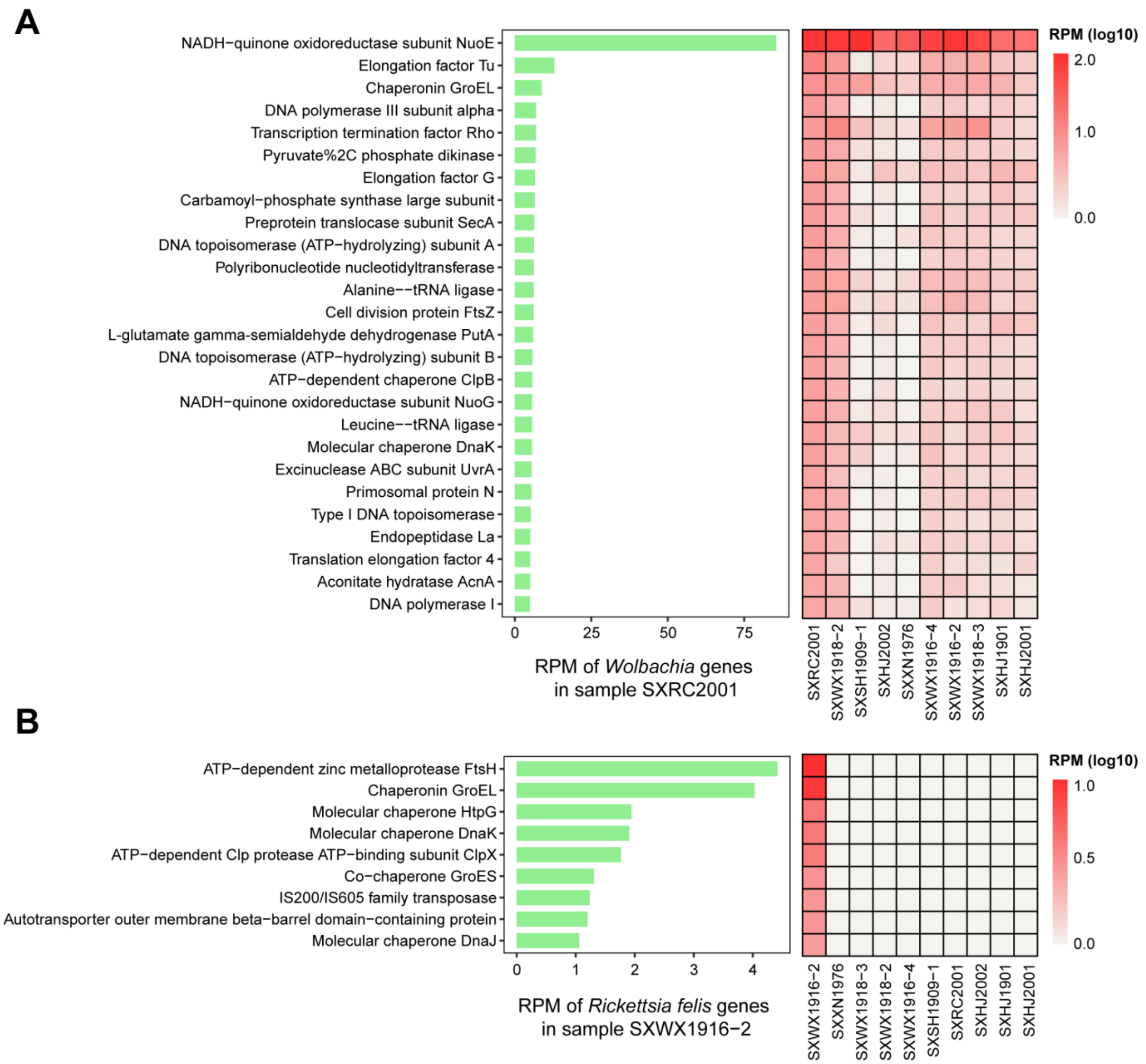

Fig. S6 Transcriptomic profiles of *Wolbachia* and *Rickettsia*

(A) Gene expression abundance of *Wolbachia* in SXRC2001 (left) and transcriptomic profiles in the ten pools (right). (B) Gene expression abundance of *Rickettsia* in SXWX1916-2 (left) and transcriptomic profiles in the ten pools (right).
